# Network Connectivity in Epilepsy: Resting State fMRI and EEG–fMRI Contributions

**DOI:** 10.3389/fneur.2014.00093

**Published:** 2014-07-04

**Authors:** Maria Centeno, David W. Carmichael

**Affiliations:** ^1^Imaging and Biophysics Unit, Institute of Child Health, University College London, London, UK; ^2^Epilepsy Unit, Great Ormond Street Hospital, London, UK

**Keywords:** epilepsy, functional connectivity, EEG–fMRI, resting state, resting state networks, RS-fMRI

## Abstract

There is a growing body of evidence pointing toward large-scale networks underlying the core phenomena in epilepsy, from seizure generation to cognitive dysfunction or response to treatment. The investigation of networks in epilepsy has become a key concept to unlock a deeper understanding of the disease. Functional imaging can provide valuable information to characterize network dysfunction; in particular resting state fMRI (RS-fMRI), which is increasingly being applied to study brain networks in a number of diseases. In patients with epilepsy, network connectivity derived from RS-fMRI has found connectivity abnormalities in a number of networks; these include the epileptogenic, cognitive and sensory processing networks. However, in majority of these studies, the effect of epileptic transients in the connectivity of networks has been neglected. EEG–fMRI has frequently shown networks related to epileptic transients that in many cases are concordant with the abnormalities shown in RS studies. This points toward a relevant role of epileptic transients in the network abnormalities detected in RS-fMRI studies. In this review, we summarize the network abnormalities reported by these two techniques side by side, provide evidence of their overlapping findings, and discuss their significance in the context of the methodology of each technique. A number of clinically relevant factors that have been associated with connectivity changes are in turn associated with changes in the frequency of epileptic transients. These factors include different aspects of epilepsy ranging from treatment effects, cognitive processes, or transition between different alertness states (i.e., awake–sleep transition). For RS-fMRI to become a more effective tool to investigate clinically relevant aspects of epilepsy it is necessary to understand connectivity changes associated with epileptic transients, those associated with other clinically relevant factors and the interaction between them, which represents a gap in the current literature. We propose a framework for the investigation of network connectivity in patients with epilepsy that can integrate epileptic processes that occur across different time scales such as epileptic transients and disease duration and the implications of this approach are discussed.

## Introduction

The notion of networks in epilepsy has gained momentum in the last decade, becoming a key concept used to explain the phenomena observed in this condition. Seizure generation, spread and termination as well as therapeutic response and cognitive impairment may be explained by the interactions between, and dysfunction of, large-scale networks. Early evidence for the involvement of macroscopic networks in epilepsy syndromes arises from EEG studies (1) and, for the last decade, several authors have developed a framework based on brain networks to explain various features of epilepsy (2–5). There is a growing body of evidence pointing toward large-scale networks, often bihemispheric and involving several lobes, underlying seizures in different epileptic syndromes (5).

Imaging studies have been one of the main contributors to the development of this network framework and have provided relevant information for the characterization of macroscopic network abnormalities in the epileptic brain. Functional MRI is a powerful tool to investigate connectivity and organization of brain networks via differences in evoked responses to different stimuli. Resting state fMRI (RS-fMRI) has become an increasingly popular way to employ fMRI that investigates synchronous activity between regions in the absence of an explicit task based on signal correlation. These studies have shown that there is a consistent pattern of spatially distinct, brain networks that show coherent signal fluctuations. RS-fMRI studies have been used to identify network abnormalities in many different pathologies including epilepsy (6).

Different approaches have been applied to the investigation of network connectivity in RS-fMRI studies. The first most commonly used methodology is seed-based correlation maps (7), where the correlation between *a priori* defined regions of interest (ROI) is calculated within a temporal frequency range and used as an index of connectivity. Regions can contain common variance from various noise sources and this need to be removed, for example via regression or partial correlation (8, 9). This approach can be extended by using an anatomical parcelation of the brain from the lobar to the voxel scale and correlating every region with all other regions before comparing the resulting correlation matrix. These matrices represent a measure of the whole brain connectivity (connectome) and can be thresholded and binarized to obtain summaries of network properties using graph theory such as clustering, path length, and betweenness centrality. Each of these metrics has well characterized implications for networks in terms of properties such as their efficiency for information transfer and robustness to damage.

Several metrics have been used to look at measures of correlation with some spatial support; these include methods such as regional homogeneity (ReHo) (10), functional integration (11), and global brain asymmetry.

The second main method is spatial independent component analysis of fMRI data that separates the signal into spatial maps of covarying voxels (12). Components that are related to brain activity then need to be identified as resting state networks by selecting them from components related to sources of noise. This is typically done by looking at the spatial and temporal properties of the components.

Brain network connectivity is not static and so the investigation of network dynamics is an important further step. Not only correlations, but causality between nodes can be evaluated. This has been achieved for example through biophysical computational models such as dynamic causal models (DCM) (13), structural equation modeling (14), and granger causality (15). Although some methods have shown their robustness on modeling causal statistical influences between simultaneously recorded neural time series data (16), the use of these methods in fMRI data is still controversial. This is due to the inherent limitations of fMRI: slow dynamics, regional variability of the hemodynamic response to underlying neuronal activity and the complexities of image acquisition (differences in slice timing). These methods must be used with care and with an appropriate understanding of their limitations (17–19).

Not only analysis methodology but also the definition of rest is different across RS-fMRI studies. Subjects may be instructed to rest with the eyes closed or open with or without visual fixation and these may be a confounding factor; in epilepsy drowsiness may be associated with a different rate of interictal activity and this may be influenced by the instructions given to the subject.

More detail information on the history, development of methods and limitations of RS-fMRI studies can be found in these reviews (6, 20). The development of simultaneous EEG–fMRI acquisition has enabled a major step to be taken toward the identification of network abnormalities related to epileptic activity. Detailed information about the methodology and its evolution can be found in recent reviews (21–23).

EEG–fMRI can be thought of as an extension of RS-fMRI, where the lack of a model of fMRI changes defined by an experimental paradigm is replaced by a *post hoc* electrophysiologically defined model of brain state. In studies of epilepsy, this is typically achieved by defining epochs of pathologic (e.g., interictal epileptiform discharges or ictal activity) versus normal background activity, although a similar approach can be applied to derive a model from physiological rhythms (e.g., alpha and beta) (24, 25). A voxel-wise analysis can then proceed to identify the brain regions with fMRI changes associated with these electrophysiological features.

Early application of EEG–fMRI was aimed at better characterization and more accurate localization of the brain areas involved in interictal spike generation (26), but it soon evolved into a research tool capable of investigating brain function in diseased and healthy populations.

However, it became clear that EEG–fMRI studies often revealed networks commonly reported in RS-fMRI studies (27). Despite this commonality and potential convergence of results there is very little cross reference between studies looking into brain networks at rest in epilepsy and EEG–fMRI studies, making it timely to comparatively summarize the findings from each strand of literature. One of the key questions that arise is regarding the role that epileptic transients might play in the findings of RS-fMRI studies, where this factor has been largely neglected.

The interaction between these two (network connectivity and epileptic transients) may be of a bidirectional nature. While the often transient stochastic nature of interictal and ictal discharges might imply a transition between (bi-stable) states it seems likely that these events occur in patients because of an alteration in network properties that facilitates abnormal synchronization within and between brain regions (or makes these transitions more likely by altering the systems dynamics). The properties of the epileptic network seem to evolve over multiple timescales, indexed by the typical clinical observations that epileptic events frequency is modulated by cognitive load, sleep, stress, and disease duration. Therefore, we need to better understand and evaluate network structure in epilepsy and the dynamic changes occurring within it at multiple timescales (from milliseconds to years).

Since some RS-fMRI studies have found an association between network abnormalities and clinical variables that in turn often correlate with frequency of epileptic transients (i.e., age of epilepsy onset, duration of epilepsy, response to treatment, and cognitive function), it makes sense to consider that the integration of this information in the investigation of network abnormalities in epilepsy will lead to novel ways of interpreting network changes observed.

**Figure 1:**
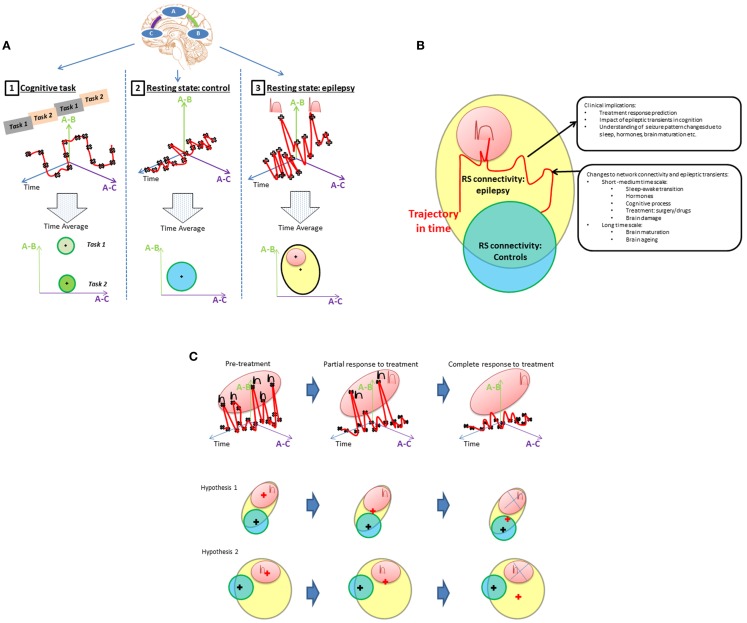
**(A)** Representing state dependant connectivity. If we consider a simple brain network with three linearly connected nodes (top), then connectivity between pairs of regions **(A,B)** or **(A,C)** can be graphically represented as a function of time (upper row graphics-red line). During: (1) cognitive tasks; (2) resting state in healthy population; and (3) resting state in patients with epilepsy. The fluctuations of this network’s connectivity in time could be measured at points illustrated by the black crosses. The brain’s connectivity state during rest and activity can then be summarized by the mean and range represented by the “+” and circles areas, respectively, in the lowest row of “Time average” plots. Epileptic transients are associated with changes in network connectivity (peaks in top right graphic) that account for a proportion of the connectivity (red area) expected to lie outside of the range associated with resting state activity in controls. The contribution of epileptic transients (red area) to the RS connectivity differences found patients with epilepsy (cross in the yellow area) and how this relates to normal connectivity (blue area in adjacent plot) still remains to be well characterized. **(B)** Connectivity differences between controls and epilepsy. Resting connectivity in patients with epilepsy (yellow area) falls out of the range (blue area) seen in controls for certain networks as reported by RS studies. Several hypotheses can be derived from this observation, e.g., (1) Connectivity changes are permanent abnormalities. (2) Connectivity changes are driven by transient epileptic activity. (3) They are a combination of both permanent and transient abnormalities. There are a number of factors that are know to modify connectivity and frequency of epileptic transients at different time scales: from cognitive process, sleep/awake transitions, or treatment effects in the short–medium term through to brain maturation and aging occurring at a long term scale. An example change in connectivity due to such factors is shown as trajectory in time (red line) through a space defined by the connectivity that can be measured using RS-fMRI. However, to understand connectivity changes associated with these factors, it is crucial to obtain measurements at different time points along the trajectory and associated them with clinically relevant factors. If RS connectivity dynamics and the role of epileptic transients in altering measured RS-fMRI connectivity are understood, RS connectivity measurements may be a potential biomarker of a number of clinically relevant aspects in epilepsy such as prediction of response to treatment, cognitive dysfunction associated to epilepsy or change to seizure patterns due to hormones, sleep, brain maturation, etc. **(C)** Example of a model of connectivity changes applied to investigate drug treatment in epilepsy. Any factor that modifies the rate of epileptic transients will result in changes to the RS connectivity. In the case of medical treatment, different degrees of response (partial or complete) would be associated with different connectivity states in a patient; more epileptic transients, means the network would spend more time with connectivity values in the “epileptic transient connectivity region” (red area) as illustrated in the first row graphic. How these changes to connectivity affect the mean connectivity of a patient with epilepsy is dependant on the proportion of abnormal connectivity explained by the epileptic transients. In this case, two scenarios are possible. Hypothesis 1: connectivity abnormalities in patients with epilepsy are mainly due to the abnormalities associated to epileptic transients, in which case, the gradual reduction of transients in time will result in the mean connectivity of a patient with epilepsy (represented by a red +) progressively moving towards connectivity found within the healthy population (represented by the blue circle with the mean on the black cross position). Alternatively, hypothesis 2 illustrates how if only a proportion of connectivity abnormalities are due to epileptic transients, connectivity may change in time due to treatment with a reduction in epileptic transients, however, the connectivity remains significantly different to the healthy population with potential therapeutic and cognitive consequences.

The integration of EEG information into network connectivity analysis requires the dynamic of connectivity to be considered. Classically, connectivity studies have assumed network connectivity can be characterized as the mean over a period of time (an fMRI session). This is represented in Figure 1A. Inferences about network connectivity differences between patients and controls have been estimated by comparing average connectivity. The addition of the temporal dimension (Figure 1B) opens the door to explore the interaction between connectivity and epileptic activity.

In this review, network abnormalities reported by RS-fMRI and EEG–fMRI studies are compared side to side and the role of epileptic transients in the RS-fMRI findings to date is discussed. Finally, we propose and discuss a framework to investigate the interaction of epileptic transients in connectivity (Figures 1A,B) and the potential applications of this framework (Figure 1C).

## Epileptogenic Network Abnormalities

The epileptogenic network refers to the areas involved in generation and spread of epileptic activity. These networks may vary across the different syndromes. Epilepsy syndromes have traditionally been classified based on the electro-clinical patterns, into focal and primary generalized syndromes (28). Focal epilepsies are defined by EEG correlates circumscribed to an area of the cortex as opposed to generalized syndromes in which the totality of the cortex is thought to be involved in seizure generation.

Although this classical view marks a clear difference based on the extent of cortex involved in seizure generation, there is a growing body of evidence pointing toward the involvement of large-scale networks underlying both the focal (5, 29) and the generalized syndromes (30–33) as well as evidence of epileptic activity being focally initiated in idiopathic generalized epilepsy (IGE) (29, 32). From this perspective, the boundaries between “focal” and “generalized” epilepsies have become more blurred. Under this framework, the concept of zones (e.g., the epileptogenic zone), adopted from the stand point of epilepsy surgery (34) can become more general in meaning. The epileptogenic zone and seizure onset zone could be exchanged for the network nodes that (by removal) can alter the network properties such that seizures cannot be generated. The network framework makes the potential range of processes and mechanisms of seizure generation and spread more varied; seizures arising from a hyper excitable region may entrain a larger neural network (5). Furthermore, recent theoretical studies of networks suggests that the network structure itself can generate seizures with or without the hyper excitable region (29).

Resting state fMRI studies in patients with epilepsy have provided extensive information about abnormalities in the epileptogenic networks in the different epileptic syndromes (Table 1).

**Table 1:** **Resting state studies in epilepsy reporting abnormalities of the epileptogenic network**.

Syndr.	Seed ROI	Connectivity findings			Method	Analysis	*N*	Effect spikes	Correlations	Reference
		Decrease	Increase	Other						
TLE	Hippocampus Thalamus	From hippocampus:Superior medial gyrusMidcingulate gyrusContralateral posterior cingulate (DMN)From thalamus: IFG	From hippocampus Parietal lobe Middle temporal gyrus		Seed ROI	P vs. CTR Correlation with structural abnormalities	15 P 15 CTR	No		Holmes et al. (115)
TLE	Hippocampus				Seed ROI	Correlation with memory scores	15 P 15 CTR	No	Memory scores positive correlation with connectivity to contralateral hippocampus and negative correlation with ipsilateral hip	Holmes et al. (55)
mTLE	Amygdala Hippocampus	DMN Contralateral mTL Limbic prefrontal regions			Seed ROI	P vs. CTR	23 P 23 CTR	Yes Simultaneous EEG–fMRI Excluded sessions with IED		Pittau et al. (42)
mTLE+ HS	Hippocampus	DMN angular gyri, thalami medial frontal			Seed ROI correlation	P vs. CTR Correlation with memory scores	21 P 12 CTR	No	RTLE: increased connectivity to frontal regions, better performanceLTLE: increased connectivity to posterior regions – worse performance	Doucet et al. (116)
mTLE	Hippocampus			Left hippocampus influences right	Granger causality	P vs. CTR Correlation with duration/age onset	19 P	No	Epilepsy duration above 10 years correlates: increases of inter-hippocampal connectivity Swap of directionality of influence	Morgan et al. (41)
TLE	Hippocampus Amygdala Entorhinal c. Brodmann 38	TL network epileptic side	TL network contralateral side	IC EEG connectivity pattern is opposed to fMRI connectivity pattern	Seed ROI	Comparison between ipsi-contralateral networkIC EEG connectivity vs. fMRI connectivity	5 P	No		Bettus et al. (38)
mTLE	Hippocampus Amygdala Entorhinal c. Brodmann 38	TL network epileptic side	TL network contralateral side		Seed ROI	P vs. CTR Correlation with clinical factors Correlation with cognitive scores	22 P 36 CTR	No	No correlation with clinical data (*N* seizures/disease duration/onset) but increases correlated with cognitive scores	Bettus et al. (36)
mTLE	Hippocampus Amygdala Entorhinal c. Brodmann 38	TL network epileptic side	TL network contralateral side		Seed ROI	P vs. CTR Correlation with cognitive scores	8 TLE26 CTR	No	Increases on connectivity correlates with memory performance	Bettus et al. (37)
mTLE+ HS	Hippocampus	Ipsi-contralateral Hippocampus			Seed ROI	P vs. CTR	18 P 9 CTR	No		Pereira et al. (39)
Focal	EEG–fMRI activation within resection area				Seed ROI	Correlation with surgical outcome	18 P 14 CTR	No	Strongly lateralized connectivity map correlates with good surgery outcome	Negishi et al. (117)
Focal (nodular heterotopia)	Heterotopic nodule/s			Network composed by other nodules and overlying cortex	Seed ROI	Correlation with epilepsy duration Correlation with tractography	11 P	No	Longer duration of epilepsy correlates with greater connectivity abnormalities Functional connectivity maps correlate with tractography	Christodoulou et al. (118)
Focal/IGE	Global brain connectivity 45 Homologous ROI	Interhemispheric coherence	Global asymmetry in temporal and limbic networks		Global c.-asymmetryFunctional integration	P vs. CTR Focal vs. Gen ep	100 P 80 CTR	No		Zhang et al. (11)
Focal	Global brain connectivity Voxel-by-voxel		Increase connectivity epileptogenic zone	Good concordance with other localizing methods	Global c.- Voxel-wise connectivity	P vs. CTR	6 P 300 CTR	No		Stufflebeam et al. (48)
TLE	Global brain connectivity Voxel-by-voxel	All group Cerebellum EEG-spikes (6) EEG-non-spikes Right medial frontal gyrus Cerebellum	All groupRight mTLDMNEEG-spikes (6)Right fusiform gyrusDMNEEG-non-spikesRight inferior temporal gyrusDMN		Global c.- ReHo	P vs. CTR Interictal vs. not interictal activity	21 P 21 CTR	Yes (deferred EEG)P with vs. P without interictal EEG activity		Mankinen et al. (47)
mTLE	Global brain connectivity 90 ROI	Frontal lobe Parietal lobe DMN	Medial temporal lobe	Altered small world network properties	Global c.- Graph t.	P vs. CTR	18 P 27 CTR	No		Liao et al. (95)
IGE (CAE)	16 ROI in epileptic network		Lateral orbito-frontal cortex interhemisphere		Global c.- Seed ROI	P vs. CTR	16 P 16CTR			Bai et al. (52)
IGE	Thalamus Dorsal nucleusLateral nucleusPulvinar nucleus	Orbito-frontal Caudate Putamen			Seed ROI	P vs. CTR VBM correlation	52P 67 CTR	No	Correlation with atrophic areas/VBM	Wang et al. (51)
IGE	Basal ganglia network	SMA Cerebellum	Basal ganglia		ICA	P vs. CTR IED vs. non-IED sessions	29 P 25 CTR	Yes IED sessions vs. non-IED sessions		Luo et al. (49)
IGE	90 ROI	Nodal topological characteristics DMN	Nodal topological characteristics mesial frontal cortex, putamen, thalamus amygdala		Global c.- Graph t.	P vs. CTR Structural connectivity vs. functional connectivity	26 P 26 CTR	No	Decoupling between structural and functional connectivity correlates with epilepsy duration	Zhang et al. (106)
IGE (CAE)	Voxel-by-voxel Seed ROI Precuneus Thalamus	Basal ganglia Precuneus to thalamus	Precuneus		Global c.-Voxel-wise connectivitySeed ROI	P vs. CTR	11 P CTR	Yes	Additional correlation with sleep	Masterton et al. (50)

*For each study, information is provided regarding the epileptic syndrome included in the study, the areas where connectivity was seeded from (ROI), in case of those studies using this approach; the main findings subdivided in increases and decreases of connectivity, and whether the effect of the spikes was addressed in the study (effect of spikes), as well as the correlations if any of the findings with clinical data*.

*Synd., epileptic syndrome; Seed ROI, region of interest used as the connectivity seed; P, patients; CTR, controls; Focal, focal epilepsies; TLE, temporal lobe epilepsy; mTLE, medial TLE; HS, hippocampal sclerosis; IGE, idiopathic generalized epilepsies; CAE, childhood absence epilepsy; IDE, interictal epileptiform discharges; ICA, independent component analysis; Global c., global brain connectivity; Graph t., graph theory; ReHo, regional homogeneity; ALFF, amplitude of low-frequency fluctuations*.

The majority of RS-fMRI studies in focal epilepsies have focused on temporal lobe epilepsy (TLE). TLE has the advantage of being one of the most prevalent and homogeneous group within the focal epilepsy syndromes, and although it provides a good model for investigating abnormalities in the epileptogenic network, the impact of these findings for surgical management of patients is limited given the efficacy of standard pre-surgical evaluation and surgical approaches in this group (35).

The epileptogenic network in TLE is relatively well characterized (5), comprising of a number of structures in the mesial temporal lobe (amygdala and hippocampus), adjacent cortex including entorhinal cortex and lateral temporal cortex and extra temporal structures including thalamus and orbito-frontal cortex. The contralateral homologous regions serve the rapid spread of seizure activity. Connectivity maps seeding in these areas of the epileptogenic network have shown a number of abnormalities, comprising decreased connectivity within a set of sub regions in the epileptic temporal lobe (36–39), decreased connectivity between hippocampi (39–42), and decreased connectivity between the hippocampus and the orbito-frontal region (40). Decreased connectivity is the most common finding among those studies targeting the epileptogenic network; hence it is interesting to compare these findings with other measures of neuronal connectivity such as EEG. Although not recorded simultaneously, intracranial EEG (icEEG) showed an increase in connectivity between the same subset of regions found to be less connected by fMRI (38). Connectivity of the epileptogenic regions measured by EEG has shown diverse results. Classically, hyper-synchrony within the epileptogenic regions has been shown using intracranial electrodes (43, 44), however, evidence of decreased synchronization of electrical activity have also been reported during ictal (45) and interictal (46) states. Variability of connectivity between studies in the epileptogenic regions may be in part explained by the difference in methodology between studies (type of intracranial electrodes, the analysis method applied to the data and the regions included in the network). However, the investigation of temporal dynamics of EEG-based connectivity shows that the desynchronization in the epileptogenic regions fluctuate at different time points (46).

The relationship between network connectivity as measured by EEG and fMRI has barely been explored. Recently, we introduced a framework to investigate this in EEG–fMRI data acquired simultaneously (Deligianni et al., submitted). In this work, we showed that EEG-based connectivity had more intra-hemispheric components compared with MRI-based connectivity that showed a predominance of inter-hemispheric connections. The prediction of connectivity patterns from one modality to the other worked better when fMRI is predicted from EEG than vice versa, indicating that EEG connectivity may have a greater level of complexity compared to that derived from fMRI.

Further research is needed to be done in order to further understand the relationship between the connectivity measured by these two modalities and in turn to interpret the similarities and discrepancies seen in the pathological brain.

Although the majority of RS-fMRI studies report decreases of connectivity within the epileptogenic network, there are also reports of increased connectivity. These increases may be located in areas overlapping the epileptogenic region, but are typically reported in areas outside the epileptogenic region suggesting a compensatory mechanism: Bettus et al. reported in several studies that connectivity was increased in the homologous network contralateral to the disrupted epileptogenic network in TLE, which are known to be propagation areas (36, 37).

Whole brain connectivity analysis have found abnormalities in areas belonging to the epileptogenic network: using ReHo analysis of fMRI as a measure of abnormal local synchronicity, Mankinen et al. (47) have reported abnormalities in the right temporal lobe and default mode network (DMN) areas in a sample of patients with left and right non-lesional TLE. In another voxel-by-voxel analysis of local and long distance connectivity, Stufflebeam et al. (48) have shown abnormalities co-localized with the epileptogenic zone defined by icEEG in patients with focal epilepsy of different locations.

Similarly, in patients with IGE, several studies have investigated abnormalities in the epileptogenic networks by creating connectivity maps from areas found to be involved in the seizure generation in these syndromes. The thalamus and the basal ganglia are most commonly chosen as seed regions, and they typically show reduced connectivity with other components in the network, mainly subcortical structures and orbito-frontal cortex (49–51). There are also reports of increased connectivity between hemispheres as shown by Bai et al. (52) in the lateral aspect of the orbito-frontal cortex in patients with childhood absence epilepsy (CAE) using two independent connectivity analysis methods (ROI seed maps and a voxel-by-voxel approach).

Methods that do not use *a priori* spatial hypothesis such as ICA (49, 53), voxel-by-voxel connectivity analysis (50), or ReHo analysis (54) have also pointed to the existence connectivity abnormalities in the so called cortico-subcortical network. In the study by Moeller et al. (53), the component corresponding to the cortico-subcortical network was found to be highly correlated with the interictal activity recorded simultaneously providing strong evidence that the epileptiform transients play a key role in the connectivity abnormalities uncovered by RS-fMRI in IGE.

Just a few studies have investigated how connectivity changes relate to clinical factors. Morgan et al. (41) showed that the initial disruption of inter-hippocampal connectivity evolves into an increased connectivity after 10 years of disease duration in TLE. Directionality of the hippocampal influence also changes with the duration of epilepsy; in general there is a left over right hippocampal influence, regardless the side of epilepsy, however, this relationship is switched in patients with epilepsy duration >10 years where the contralateral hippocampus has the dominating influence over the affected one.

Connectivity changes have been also related with memory function in TLE: memory scores are preserved in those patients with stronger connectivity to the contralateral temporal lobe (55) and in those with stronger intra-hippocampal connectivity and between hippocampus and frontal areas; and conversely, decreased connectivity to the orbito-frontal cortex was related to poorer memory performance (40).

EEG–fMRI was conceived as a technique to map the epileptogenic networks. In focal epilepsies, several studies have shown found a good concordance between the regions of BOLD signal change during interictal activity and the epileptogenic regions mapped by standard techniques (56–63). It has been estimated that EEG–fMRI can contribute to more accurately localize the epileptic focus in around 2/3 of pre-surgical cases as compared to the standard pre-surgical tests (60).

Similarly, in IGE, a large number of EEG–fMRI studies have characterized the networks involved in the generation of epileptic activity (64–68). Common findings across studies show activation of a cortico-subcortical network composed by mid-frontal regions, thalami, caudate, and cerebellum during the occurrence of generalized spike-waves.

From early EEG–fMRI studies, it was noted that responses are often multiple and distributed in areas within the epileptic focus but also remotely located from epileptogenic regions in the case of focal epilepsies. Changes in BOLD signal have been reported on the contralateral homologous cortex, as well as extra temporal regions in patients with TLE (56, 58) and (predominantly negative) responses in DMN areas (27, 66). This supports the presence of large-scale, often bilateral networks underlying focal epilepsies and the involvement of other networks such as the DMN during epileptic activity.

There is on-going work aimed at objectively deriving the epileptogenic zone from EEG–fMRI maps in order to provide information that can be used for surgical evaluation (69). Different authors have chosen different statistical methods for its definition: from the global maxima of response (59), to the number of voxels within the cluster (70). Several methods have aim to separate regions of propagation from those involved in initiation such as electrical source imaging (ESI) (71, 72) of interictal spikes. These methods have been tested against surgical outcome, which is the gold standard for the localization of the epileptogenic network and more importantly assessing clinical utility. Good surgical outcome has been associated with the inclusion of the global maxima of response being within the resection margins (59). On the contrary, responses discordant with the area of surgery, and widespread responses are a marker of poor prognosis (59) this was also observed in a group of patients with focal cortical dysplasia (confirmed post-resection) whose post-surgical prognosis is typically good (73) likely indicating multifocal disease (74, 75).

The dynamics of epileptic networks in focal epilepsy (76, 77) and in IGE (30, 78) have been investigated using DCM and sliding window analysis, aiming to identify the temporal and causal relationship between network nodes.

In IGE, crucial subcomponents of the network such as the thalamus (79) have been targeted to further define their role, which could potentially inform targets for future therapies such as deep brain stimulation. However, there is still no consensus between the different studies as to the lead node or the exact role of the thalamus. There are several factors that might explain these discrepant results. Firstly, there is some methodological uncertainty in the temporal relationship between generalized spike and wave discharges (GSW) and fMRI changes with several studies indicating fMRI changes can precede GSW events (31). Further, the methods used in different studies to infer causality are not consistent and neither are the network nodes. This suggest that further work is needed both from a computational perspective to better predict how GSW arise (29) and a modeling perspective to better test these predictions with experimental data (80).

Even though EEG–fMRI and RS-fMRI studies have been able to identify networks involved in the generation and spread of epileptiform activity, the interpretation of the findings greatly differs between these two approaches. Whereas EEG–fMRI studies allow inference that the changes observed are related to interictal activity, RS-fMRI cannot differentiate, which changes observed in the network may be due to transient or permanent network abnormalities. This is important, for example when trying to understand the mechanism for treatment response or effects of disease duration; is the network connectivity fundamentally altered or is it that the number of transient events and transient changes in connectivity has been reduced?

## Cognitive Network Abnormalities

Resting state fMRI studies have extensively investigated networks involved in cognitive processes and sensory-motor processing in the different epileptic syndromes (Table 2).

**Table 2:** **Resting state studies in epilepsy reporting abnormalities of cognitive networks**.

Syndr.	ROI	Connectivity findings			Method	Analysis	*N*	Effect spikes	Correlations	Reference
		Decrease	Increase	Other						
IGE		Self-referential, somatosensory, visual auditoryDMN (frontopolar/parietal)	DMN (precuneus)		ICA	P vs. CTR Correlation disease duration	16 P16 CTR	No	Disease duration correlates with medial prefrontal cortex changes in connectivity	Wang et al. (85)
TLE left	Language network	Language networks			ICA	P vs. CTR	17 P30 CTR	No		Waites et al. (81)
TLE + HS	Auditory Sensorimotor Visual networks	Auditory/sensorimotor Between visual ntw and mTL	Visual cortex		ICA	P vs. CTR Correlation with clinical factors	33 P33 CTR	No	Epilepsy duration correlate negatively with connectivity	Zhang et al. (82)
TLE + HS	Dorsal attentional network	Dorsal attentional network			ICA	P vs. CTR Correlation with cognitive scores	24 P24 CTR	No	Working memory scores correlate with connectivity in attention network	Zhang et al. (83)
IGE	18 ROI in attention network		Within attention network and adjacent areas		Seed ROI	P vs. CTR	14 P14 CTR	No	Disease duration correlates with abnormal connectivity in frontal areas	Maneshi et al. (84)
TLE	Precuneus Frontopolar	DMN Hippocampus	Left TLE to different regions	Abnormalities are epilepsy side specific	Seed ROI	P vs. CTR	23 P13 CTR	No		Haneef et al. (96)
mTLE	Precuneus Frontopolar	Hippocampus			Seed ROI	P vs. CTR Correlation with DTI	20 P20 CTR	No	Correlates fc of precuneus to mTL with DTI	Liao et al. (119)
Focal		DMN, in particular Precuneus/parietal			ICA	P vs. CTR Correlation with clinical factors	11 P11 CTR	No	No correlation with clinical factors	Widjaja et al. (98)
mTLE + HS		DMN			ICA	P vs. CTR Correlation with clinical factors	52 P29 CTR	No	Decrease connectivity in mTL structures correlate with duration	Zhang et al. (94)
TLE		RSN			ICA	P vs. CTR Interictal vs. non-interictal activity	21 P21 CTR	Yes (deferred EEG)P with IED vs. no IED	Correlation with interictal activity	Mankinen et al. (97)
Focal/IGE		Precuneus Less connected in generalized epilepsies			ICA	P vs. CTR Generalized vs. focal epilepsy	28 P34 CTR	No		Lui et al. (92)
mTLE			DMN Basal ganglia Limbic structures		Global c.- ALFF	P vs. CTRSubgroup analysis 6 P with interictal activity. Correlation of interictal spikes with ALFF	50 P25 CTR	Yes		Zhang et al. (93)
IGE	Anterior cingulate Precuneus	Prefrontal Precuneus			Seed ROI	P vs. CTR	15 P15 CTR	No	Correlation with epilepsy duration (increased connectivity PFC with parahipp and decreased connectivity PFC/PCC)	McGill et al. (99)
IGE (CAE)	Bilateral dorsal prefrontal cortexPrecuneusAnterior cingulate	DMN Cognitive control network Affective network			Seed ROI	Sessions GSW vs. sessions non-GSW	10 P	Yes	Correlation with interictal activity	Yang et al. (100)
IGE	Precuneus	DMN			Seed ROI	P vs. CTR	12 P14 CTR	Yes	Fronto-parietal connectivity correlates negatively with epilepsy duration. No correlation with other clinical variables	Luo et al. (101)
Focal/IGE	Precuneus	DMN in P with GTCS			Set functions model	Focal ep with partial sz vs. GTCS vs. CTR	28 P34 CTR	No		Lui et al. (92)
IGE		DMN			ICA	P drug resistant vs. P drug responsive vs. CTR	60 P38 CTR	Yes	Correlates with drug resistancy	Kay et al. (114)
IGE		DMN			ROI/Graph t.	P vs. CTR	14 P29 CTR	No		Song et al. (102)

*For each study, information is provided regarding the epileptic syndrome included in the study, the areas where connectivity was seeded from (ROI), in case of those studies using this approach; the main findings subdivided in increases and decreases of connectivity, and whether the effect of the spikes was addressed in the study (effect of spikes), as well as the correlations of the findings with clinical data if any*.

*Synd., epileptic syndrome; Seed ROI, region of interest used as the connectivity seed; P, patients; CTR, controls; Focal, focal epilepsies; TLE, temporal lobe epilepsy; mTLE, medial TLE; HS, hIPPOCAMPAL sclerosis; IGE, idiopathic generalized epilepsies; CAE, childhood absence epilepsy; IDE, interictal epileptiform discharges; Global c., global brain connectivity; Graph t., graph theory; ReHo, regional homogeneity; ALFF, amplitude of low-frequency fluctuations*.

Abnormalities include decreased connectivity in language network (81), memory network (40), auditory and sensorimotor networks (82) as well as increases in connectivity of visual and dorsal attention networks (83) in patients with focal epilepsies. In IGE in whom cognition is expected not to be grossly abnormal, increased connectivity was found within the nodes of the attention network and between attention network and adjacent the supplementary motor area (84). Also self-referential, somatosensory, visual, and auditory networks connectivity is increased in IGE patients compared to controls (85).

In the case of cognitive networks, there is a higher variability between the changes observed as both reports of increases and decreases in connectivity are found in the literature in similar numbers.

The increases in connectivity observed in some studies have been associated to efficient compensatory changes that maintain cognitive function (40, 83), but there are also notable reports of poorer function associated with the abnormal increase in connectivity between networks. For example, in juvenile myoclonic epilepsy (JME) increased SMA and working memory network functional connectivity was linked with increased demands in working memory function (86). This finding offered an explanation for the myoclonic jerks associated with cognitive-motor tasks that are found in this syndrome. Similarly, in patients with TLE, increased connectivity of working memory networks to the diseased hippocampus was associated with poorer performance in working memory tests (87).

How epileptic transients may affect these RS-fMRI findings is uncertain due to the lack of studies investigating this potential influence. In a report by Chaudhary et al. (88), EEG activity was monitored during a working memory–fMRI session; task related activation was found to be significantly decrease during the epileptic transient period. Interestingly, a modulatory effect of the task was also found on the frequency of epileptic activity that in turn was associated with task performance. In reflex epilepsies, the interaction between epileptic activity and cognitive network connectivity becomes even more pertinent, Vaudano et al. (76) showed in a patient with reading epilepsy, that areas within the cognitive network involved in reading (left prefrontal cortex) played a causal role in initiating reading-evoked seizures, potentially by facilitating activity in the epileptogenic cortex, in this case, located in the premotor cortex. These reports again show the need for the application of EEG information to better understand connectivity changes within and between cognitive networks in patients with epilepsy.

Abnormalities in the DMN deserve special attention due to the extended literature in this regard both from RS-fMRI and EEG–fMRI studies. In relation to interictal activity in focal epilepsies, EEG–fMRI studies have found BOLD signal changes in DMN (27, 89) with differences in the strength and pattern between TLE and extra-TLE (27, 89). DMN BOLD changes are also common to patients with IGE (64–68). These studies, across focal and IGE, point predominantly to a decrease of BOLD signal in DMN during epileptic transients.

A recent study (90) has provided relevant insights about the electrophysiological correlates of this phenomena: a decrease of gamma power and increase of lower frequencies, occurs synchronously with interictal activity in the main nodes of DMN when recorded with icEEG. This may explain the negative change in BOLD signal found in these areas coupled with epileptic activity, and confirms that the coupling between the BOLD and EEG signals remains intact (91).

Although there have been many studies finding DMN alterations in epilepsy there remains large gaps in our understanding of the interaction between the epileptogenic network and the DMN. Interestingly, a study using effective connectivity (30) showed that this response in the precuneus was predictive of changes within the thalamo-cortical regions. This is consistent with the idea that conscious attention (indexed by the precuneus) modulates the connectivity of the thalamo-cortical loop and can therefore alter the probability of GSW generation.

Resting state fMRI studies have extensively investigated DMN and have consistently reported abnormal connectivity within the DMN and between the DMN and epileptogenic regions in focal epilepsies (92–98) and IGE (49, 50, 85, 92, 99–102). The most common finding is a decrease in the connectivity within DMN and between the epileptogenic regions with DMN. However, there are also some reports of increased connectivity in certain nodes like the precuneus (50, 85).

The correlation between the DMN and functioning of other cognitive networks in fMRI (103) and its proven strong correlation with the epileptic activity points toward the need to test cognitive networks abnormalities in epileptic patients in light of the EEG information.

## Altered Global Brain Connectivity

Mathematical tools to derive global network organization such as graph theory have been applied to fMRI (and more rarely EEG) data to identify abnormalities in patients with epilepsy. In this section, we will discuss the changes in global network organization found in patients with epilepsy (Table 3).

**Table 3:** **Resting state studies in epilepsy reporting abnormalities of global brain connectivity**.

Syndr.	ROI	Connectivity findings			Method	Analysis	*N*	Effect spikes	Correlations	Reference
		Decrease	Increase	Other						
FLE	Global brain connectivity	Long range connections	Interhemispheric connections	Increased modularity in patients	Global c.- Graph t.	P vs. CTR Correlation	37 P41 CTR	No	Increased modularity correlates with worse cognition	Vaessen et al. (104)
mTLE	Global brain connectivity	No specific networks		Classification of network characteristics lead to diagnostic accuracy of 77%	Global c.- Graph t.	P vs. CTR	16 P52 CTR	No		Zhang et al. (105)
Focal/IGE	Global brain connectivity	Interhemispheric coherence	Global asymmetry (temporal and limbic networks)		Global c.-AsymmetryIntegration	P vs. CTR	100P80 CTR	No		Zhang et al. (11)
IGE	Global brain connectivity	Cortical and subcortical structures			Global c.- ReHo	P vs. CTR	25 P25 CTR	No	ReHo in thalamus/insula and DMN correlated with duration of epilepsy	Zhong et al. (54)
IGE	Global brain connectivity	Nodal topological characteristicsDMN	Nodal topological characteristics mesial frontal cortex, putamen, thalamus amygdala		Global c.- Graph t.	P vs. CTRStructural connectivity vs. functional connectivity	26 P26 CTR	No	Decoupling between structural and functional connectivity correlates with epilepsy duration	Zhang et al. (106)

*For each study, information is provided regarding the epileptic syndrome included in the study, the areas where connectivity was seeded from (ROI), in those studies using this approach; the main findings subdivided in increases and decreases of connectivity, and whether the effect of the spikes was addressed in the study (effect of spikes), as well as the correlations if any of the findings with clinical data*.

Synd., epileptic syndrome; Seed ROI, region of interest used as the connectivity seed; P, patients; CTR, controls; Focal, focal epilepsies; TLE, temporal lobe epilepsy; mTLE, medial TLE; HS, hippocampal sclerosis; IGE, idiopathic generalized epilepsies; CAE, childhood absence epilepsy; IDE, interictal epileptiform discharges; Global c., global brain connectivity; Graph t., graph theory; ReHo, regional homogeneity; ALFF, amplitude of low-frequency fluctuations

Graph theory based analysis has shown that brain networks in patients with epilepsy follow a small world type topology, similar to healthy subjects. However, significant differences in the parameters that define the small world connectivity have been detected in comparison to controls: patients with focal epilepsy have an increased modularity and interhemispheric connections (104) as well as abnormal degree, strength closeness, clustering coefficient, and betweenness centrality (105). Liao et al. (95) showed these abnormalities were more marked in the epileptogenic networks and DMN of patients with TLE. In IGE, increased integration and nodularity in the cortico-subcortical network and decrease degree and nodularity of DMN nodes have been reported (102, 105).

Using global connectivity asymmetry and interhemispheric coherence as measures, patients with focal and generalized epilepsy, showed higher global asymmetry and lower interhemispheric coherence compared to controls. These abnormalities were more prominent in the temporal and limbic networks across both focal and IGE patients (11).

The clinical meaning of these findings is uncertain and only a few studies have included correlations with some clinical aspects of epilepsy such as disease duration (54, 106).

The majority of the studies applying graph theoretical analysis or voxel-wise analysis primarily set out to find differential characteristics that correctly classify patients with epilepsy from healthy control groups. Although this approach may be useful in other neurological conditions such as Alzheimer’s (107), where presymptomatic diagnosis is important, the clinical applicability is unclear in epilepsy where diagnosis is based on the occurrence of spontaneous seizures and the prediction of populations at risk remains speculative.

One of the aspects that need to be explored is the effect that the transient epileptic activity may have on these network properties, which has not yet been address by any of the studies and will provide useful information on the relation of these measures and the physiopathology of the disease. Further work is required to determine if these RS-fMRI measures can become a useful biomarker of disease progression (beyond potentially simply indexing interictal event rate) and therefore help to measure therapeutic efficacy or predict treatment response.

## Role of Interictal Activity in RS-fMRI

The effect of epileptiform activity on the networks abnormalities described in RS-fMRI studies has been largely neglected. Only a few RS-fMRI studies have included EEG information in their analysis. The most common approach has been to use the EEG to exclude the presence of interictal activity during RS-fMRI. In the absence of interictal activity on scalp EEG, Pittau et al. (42) found decreased connectivity within the DMN and in the epileptogenic network of TLE patients and similar findings have been seen in patients with IGE (49, 50). Mankinen et al. (97) reported similar findings on those patients whose EEG acquired previously to scan was showing no interictal activity, however an important limitation of this study is that presence of epileptiform activity during the scanning session cannot be ruled out due to its intermittent nature and change in prevalence in certain states (i.e., when drowsy).

Conversely, where a direct comparison between RS-fMRI sessions with and without the occurrence of spikes has been made have shown that the network abnormalities reported are more marked during the occurrence of interictal activity. In IGE, increased connectivity of epileptogenic network involving basal ganglia and decreased connectivity in DMN (101), cognitive control network (CCN) and affective network (AN) (100) were greater during those sessions with occurrence of GSW compared to those without.

Mankinen et al. (47) showed that ReHo abnormalities have a different distribution depending on the presence/absence of interictal activity on EEG acquired prior to the fMRI. Moeller et al. (53) and Rodionov (108) found that ICA can identify a component that spatially correlates with the cortico-subcortical network that is temporally correlated with epileptic transients.

There is only one RS-fMRI study where the correlation between interictal EEG activity and brain connectivity abnormalities was quantified (93). Using amplitude of low frequency oscillations (ALFF) as measure of resting connectivity in a subgroup of six patients with mTLE; increased connectivity was measured within mesial temporal lobe networks of patients, which correlated with the number of interictal events. This suggests that interictal activity was likely to be largely responsible for the network abnormalities observed.

There are two main challenges when approaching the integration of EEG information (i.e., epileptic activity) in fMRI connectivity analysis. The first limitation is scalp EEG’s sensitivity to capture epileptiform abnormalities; icEEG recordings show that only a portion of the epileptiform activity is captured by scalp EEG. This limitation needs to be taken into account in those studies that describe connectivity changes in the absence of epileptic activity monitored by scalp EEG. Acquisition of simultaneous icEEG–fMRI offers one possible solution to this sensitivity limitation (109, 110) while the extraction of scalp EEG information not visually identifiable remains another (111). The second limitation is to define the concept of abnormality in the EEG of patients with epilepsy. The classical definition of epileptiform abnormalities, useful from the clinical point of view, constrains EEG modeling to a number of abnormal features whereas EEG (and MEG) research is providing new insights into different ways of exploring and defining EEG background activity (112) and its relation to RS-fMRI derived networks (113).

Abnormalities in brain networks are likely to be present in epilepsy without visible epileptiform activity in scalp EEG as evidenced by structural connectivity changes (106, 114) but to differentiate the more permanent and transient connectivity changes might have implications; for example in understanding how treatment of the transient epileptic events might reverse their cognitive impact.

In general, to understand the sequelae of altered brain connectivity in terms of cognition and seizure likelihood, both clinically important questions, we need to disambiguate and understand the effect of brain network alterations occurring over different timescales; millisecond changes related to IEDs, tens of seconds as measured by RS-fMRI and permanent changes (e.g., measured using diffusion tensor imaging). EEG–fMRI therefore has a role to play in the functional connectivity changes occurring in the milliseconds – tens of seconds domain.

## Discussion and Conclusion

### What have we learnt from RS-fMRI and EEG–fMRI studies?

Resting state fMRI studies in epilepsy have derived information with regards to network dysfunction within and across epilepsy syndromes. In both, focal and generalized epileptic syndromes abnormalities are seen in large-scale networks usually involving more than one lobe, and with bilateral distribution. Some of the network abnormalities have common features like the disruption of DMN and the thalamo-cortical patterns seen across syndromes with spike and wave discharges (see Tables 1–3). These findings are consistent with EEG–fMRI studies, primarily modeling fMRI changes to interictal events which have also shown large-scale networks associated with epileptic activity, including changes in networks such as the DMN. However, open questions remain regarding how the RS network changes found correlate to key aspects of epilepsy such as seizure and IED generation, response to treatment (pharmacological and surgical) and cognitive dysfunction.

The strength of combined EEG–fMRI lies in the ability to define brain state and add a different range of temporal scales for assessment of dynamic changes in network activity. This allows for the identification and separation of pathologic features and their characterization.

EEG–fMRI has had some level of validation as a pre-surgical assessment tool; however it is likely to be useful in a subset of patients and requires specialist equipment and knowledge, limiting its availability to major epilepsy centers. RS-fMRI has relatively little evidence of clinical utility in pre-surgical assessment, where it needs to be predictive or diagnostic in terms of localization in individuals to have clinical impact.

### Future directions

We propose the integration of both methods as the forward step to link the abnormalities of network connectivity to the pathophysiological phenomena of the disease.

Figure 1 summarizes the questions and hypothesis derived from this review. Given the episodic nature of epileptic activity, it seems appropriate to represent functional connectivity as a dynamic trajectory through a connectivity space with time (Figure 1A). Connectivity, as indexed by fMRI correlations between regions will depend on brain state: during cognitive tasks, we expect a higher connectivity if the nodes are involved in that task (A-1 pale green area), lower connectivity if they are not (A-1, green area), and a small variability in connectivity given that cognitive processes, typically require functional segregation. In contrast, at rest, mean connectivity of that same network will be expected to be significantly different and have greater variability due to the relatively unconstrained nature of the resting state (A-2). In the case of patients with epilepsy, there is an additional component that has been found to induce changes in connectivity: epileptic transients (A-3). Meanwhile, resting state studies have determined that the mean connectivity of patients with epilepsy is abnormal, the contribution of transients to these findings is yet to be properly characterized.

In our view, the understanding of the effect of connectivity changes associated with epileptic transients on the overall RS connectivity is crucial for interpreting the findings of RS-fMRI studies to date and to understand the interaction between RS-fMRI networks in epileptic processes or cognitive co-morbidities. Current RS-fMRI studies capture connectivity changes as an average over time showing differences from controls (Figure 1B: where patient’s connectivity is represented by yellow area and controls in blue).

Epileptic transient’s rate and seizure activity may be modulated by a number of factors that occur over different timescales: from treatment to cognitive activity or external/internal factors such as hormones, sleep, or sensory stimulation. This might be because they cause changes in brain connectivity that takes them toward or away from network connectivity configuration that are associated with epileptic states (represented by the red area). One clear example is reflex epilepsies, where changes in the network involved in reading precede the seizure onset as measured by EEG–fMRI in a case with reading epilepsy (76).

To understand the effect on resting state networks of any factor of interest that interacts with epileptic transients we need to first understand their role in the global RS connectivity in epilepsy.

Taking as an example drug treatment response, we can hypothesize a change in RS connectivity based on the modification of epileptic transients due to treatment (Figure 1C): progressive decrease in epileptic activity may result in network connectivity taking values that fall outside the “epileptic transient connectivity zone” and in turn that are more similar to controls connectivity. The characterization of these changes may allow RS-fMRI results to be used as a marker of relevant aspects of the disease at different time scales: such as response to treatment, cognitive effects of epilepsy, transition between interictal and ictal states, or chronic effects of disease progression.

Resting state fMRI and connectivity analysis is a fast developing field of research and is therefore set to benefit from substantial methodological advances with faster data acquisition, reduced artifacts and improved and better validated analysis procedures.

Future work needs to ground results in clinically observed features such as the change in epileptiform activity, or seizure rates over different timescales, e.g., with different levels of attention, in different sleep states, or over months or years of disease progression. Therefore, allowing us to better understand how changes in brain networks occurring over different timescales contribute to the clinical manifestations of epilepsy and their control. While RS-fMRI provides an important non-invasive tool to evaluate network structure in epilepsy the addition of EEG recording should allow for better inference regarding the dynamic changes occurring at multiple timescales in epilepsy.

## Conflict of Interest Statement

The authors declare that the research was conducted in the absence of any commercial or financial relationships that could be construed as a potential conflict of interest.

## References

[1] Gloor P Generalized cortico-reticular epilepsies. Some considerations on the pathophysiology of generalized bilaterally synchronous spike and wave discharge. Epilepsia (1968) 9(3):249–63 10.1111/j.1528-1157.1968.tb04624.x 4975028

[2] Laufs H Functional imaging of seizures and epilepsy: evolution from zones to networks. Curr Opin Neurol (2012) 25(2):194–200 10.1097/WCO.0b013e3283515db9 22322414

[3] Halasz P The concept of epileptic networks. Part 2. Ideggyogy Sz (2010) 63(11–12):377–84 21409869

[4] Halasz P The concept of epileptic networks. Part 1. Ideggyogy Sz (2010) 63(9–10):293–303 21033313

[5] Spencer SS Neural networks in human epilepsy: evidence of and implications for treatment. Epilepsia (2002) 43(3):219–27 10.1046/j.1528-1157.2002.26901.x 11906505

[6] Snyder AZ Raichle ME A brief history of the resting state: the Washington University perspective. Neuroimage (2012) 62(2):902–10 10.1016/j.neuroimage.2012.01.044 22266172 PMC3342417

[7] Biswal B Yetkin FZ Haughton VM Hyde JS Functional connectivity in the motor cortex of resting human brain using echo-planar MRI. Magn Reson Med (1995) 34(4):537–41 10.1002/mrm.1910340409 8524021

[8] Fox MD Snyder AZ Vincent JL Corbetta M Van Essen DC Raichle ME The human brain is intrinsically organized into dynamic, anticorrelated functional networks. Proc Natl Acad Sci U S A (2005) 102(27):9673–8 10.1073/pnas.0504136102 15976020 PMC1157105

[9] Smith SM Miller KL Salimi-Khorshidi G Webster M Beckmann CF Nichols TE Network modelling methods for FMRI. Neuroimage (2011) 54(2):875–91 10.1016/j.neuroimage.2010.08.063 20817103

[10] Zang Y Jiang T Lu Y He Y Tian L Regional homogeneity approach to fMRI data analysis. Neuroimage (2004) 22(1):394–400 10.1016/j.neuroimage.2003.12.030 15110032

[11] Zhang J Cheng W Wang Z Zhang Z Lu W Lu G Pattern classification of large-scale functional brain networks: identification of informative neuroimaging markers for epilepsy. PLoS One (2012) 7(5):e36733 10.1371/journal.pone.0036733 22615802 PMC3355144

[12] McKeown MJ Makeig S Brown GG Jung TP Kindermann SS Bell AJ Analysis of fMRI data by blind separation into independent spatial components. Hum Brain Mapp (1998) 6(3):160–88 10.1002/(SICI)1097-0193(1998)6:5/6<368::AID-HBM7>3.3.CO;2-5 9673671 PMC6873377

[13] Friston KJ Dolan RJ Computational and dynamic models in neuro- imaging. Neuroimage (2010) 52(3):752–65 10.1016/j.neuroimage.2009.12.068 20036335 PMC2910283

[14] McIntosh AR Gonzalez-Lima F Structural modeling of functional neural pathways mapped with 2-deoxyglucose: effects of acoustic startle habituation on the auditory system. Brain Res (1991) 547(2):295–302 10.1016/0006-8993(91)90974-Z 1884204

[15] Roebroeck A Formisano E Goebel R Mapping directed influence over the brain using Granger causality and fMRI. Neuroimage (2005) 25(1):230–42 10.1016/j.neuroimage.2004.11.017 15734358

[16] Bressler SL Seth AK Wiener-Granger causality: a well established methodology. Neuroimage (2011) 58(2):323–9 10.1016/j.neuroimage.2010.02.059 20202481

[17] Daunizeau J David O Stephan KE Dynamic causal modelling: a critical review of the biophysical and statistical foundations. Neuroimage (2011) 58(2):312–22 10.1016/j.neuroimage.2009.11.062 19961941

[18] Friston K Moran R Seth AK Analysing connectivity with Granger causality and dynamic causal modelling. Curr Opin Neurobiol (2013) 23(2):172–8 10.1016/j.conb.2012.11.010 23265964 PMC3925802

[19] Friston K Dynamic causal modeling and Granger causality comments on: the identification of interacting networks in the brain using fMRI: model selection, causality and deconvolution. Neuroimage (2011) 58(2):303–5 author reply 10-1, 10.1016/j.neuroimage.2009.09.031 19770049 PMC3183826

[20] Cole DM Smith SM Beckmann CF Advances and pitfalls in the analysis and interpretation of resting-state FMRI data. Front Syst Neurosci (2010) 4:8 10.3389/fnsys.2010.00008 20407579 PMC2854531

[21] Rosenkranz K Lemieux L Present and future of simultaneous EEG-fMRI. MAGMA (2010) 23(5–6):309–16 10.1007/s10334-009-0196-9 20101434

[22] Gotman J Pittau F Combining EEG and fMRI in the study of epileptic discharges. Epilepsia (2011) 52(Suppl 4):38–42 10.1111/j.1528-1167.2011.03151.x 21732941 PMC3753285

[23] Laufs H Daunizeau J Carmichael DW Kleinschmidt A Recent advances in recording electrophysiological data simultaneously with magnetic resonance imaging. Neuroimage (2008) 40(2):515–28 10.1016/j.neuroimage.2007.11.039 18201910

[24] Laufs H Krakow K Sterzer P Eger E Beyerle A Salek-Haddadi A Electroencephalographic signatures of attentional and cognitive default modes in spontaneous brain activity fluctuations at rest. Proc Natl Acad Sci U S A (2003) 100(19):11053–8 10.1073/pnas.1831638100 12958209 PMC196925

[25] Moosmann M Ritter P Krastel I Brink A Thees S Blankenburg F Correlates of alpha rhythm in functional magnetic resonance imaging and near infrared spectroscopy. Neuroimage (2003) 20(1):145–58 10.1016/S1053-8119(03)00344-6 14527577

[26] Lemieux L Salek-Haddadi A Josephs O Allen P Toms N Scott C Event-related fMRI with simultaneous and continuous EEG: description of the method and initial case report. Neuroimage (2001) 14(3):780–7 10.1006/nimg.2001.0853 11506550

[27] Laufs H Hamandi K Salek-Haddadi A Kleinschmidt AK Duncan JS Lemieux L Temporal lobe interictal epileptic discharges affect cerebral activity in “default mode” brain regions. Hum Brain Mapp (2007) 28(10):1023–32 10.1002/hbm.20323 17133385 PMC2948427

[28] ILAE CoCaTotILAE. Proposal for revised classification of epilepsies and epileptic syndromes. Commission on classification and terminology of the international league against epilepsy. Epilepsia (1989) 30(4):389–99 10.1111/j.1528-1157.1989.tb05316.x 2502382

[29] Terry JR Benjamin O Richardson MP Seizure generation: the role of nodes and networks. Epilepsia (2012) 53(9):e166–9 10.1111/j.1528-1167.2012.03560.x 22709380

[30] Vaudano AE Laufs H Kiebel SJ Carmichael DW Hamandi K Guye M Causal hierarchy within the thalamo-cortical network in spike and wave discharges. PLoS One (2009) 4(8):e6475 10.1371/journal.pone.0006475 19649252 PMC2715100

[31] Moeller F Siebner HR Wolff S Muhle H Boor R Granert O Changes in activity of striato-thalamo-cortical network precede generalized spike wave discharges. Neuroimage (2008) 39(4):1839–49 10.1016/j.neuroimage.2007.10.058 18082429

[32] Meeren H van Luijtelaar G Lopes da Silva F Coenen A Evolving concepts on the pathophysiology of absence seizures: the cortical focus theory. Arch Neurol (2005) 62(3):371–6 10.1001/archneur.62.3.371 15767501

[33] Steriade M Contreras D Spike-wave complexes and fast components of cortically generated seizures. I. Role of neocortex and thalamus. J Neurophysiol (1998) 80(3):1439–55 9744951 10.1152/jn.1998.80.3.1439

[34] Lüders H General Principles of Pre-Surgical Evaluation Textbook of Epilepsy Surgery. London: Boca Raton (2008). xl, 407, 23 p.

[35] de Tisi J Bell GS Peacock JL McEvoy AW Harkness WF Sander JW The long-term outcome of adult epilepsy surgery, patterns of seizure remission, and relapse: a cohort study. Lancet (2011) 378(9800):1388–95 10.1016/S0140-6736(11)60890-8 22000136

[36] Bettus G Bartolomei F Confort-Gouny S Guedj E Chauvel P Cozzone PJ Role of resting state functional connectivity MRI in presurgical investigation of mesial temporal lobe epilepsy. J Neurol Neurosurg Psychiatry (2010) 81(10):1147–54 10.1136/jnnp.2009.191460 20547611

[37] Bettus G Guedj E Joyeux F Confort-Gouny S Soulier E Laguitton V Decreased basal fMRI functional connectivity in epileptogenic networks and contralateral compensatory mechanisms. Hum Brain Mapp (2009) 30:1580–91 10.1002/hbm.20625 18661506 PMC6870867

[38] Bettus G Ranjeva J-P Wendling F Bénar CG Confort-Gouny S Régis J Interictal functional connectivity of human epileptic networks assessed by intracerebral EEG and BOLD signal fluctuations. PLoS One (2011) 6:e20071 10.1371/journal.pone.0020071 21625517 PMC3098283

[39] Pereira FR Alessio A Sercheli MS Pedro T Bilevicius E Rondina JM Asymmetrical hippocampal connectivity in mesial temporal lobe epilepsy: evidence from resting state fMRI. BMC Neurosci (2010) 11:66 10.1186/1471-2202-11-66 20525202 PMC2890013

[40] Voets NL Adcock JE Stacey R Hart Y Carpenter K Matthews PM Functional and structural changes in the memory network associated with left temporal lobe epilepsy. Hum Brain Mapp (2009) 30(12):4070–81 10.1002/hbm.20830 19517529 PMC6870932

[41] Morgan VL Rogers BP Sonmezturk HH Gore JC Abou-Khalil B Cross hippocampal influence in mesial temporal lobe epilepsy measured with high temporal resolution functional magnetic resonance imaging. Epilepsia (2011) 52:1741–9 10.1111/j.1528-1167.2011.03196.x 21801166 PMC4428312

[42] Pittau F Grova C Moeller F Dubeau F Gotman J Patterns of altered functional connectivity in mesial temporal lobe epilepsy. Epilepsia (2012) 53:1013–23 10.1111/j.1528-1167.2012.03464.x 22578020 PMC3767602

[43] Bettus G Wendling F Guye M Valton L Regis J Chauvel P Enhanced EEG functional connectivity in mesial temporal lobe epilepsy. Epilepsy Res (2008) 81(1):58–68 10.1016/j.eplepsyres.2008.04.020 18547787

[44] Bartolomei F Chauvel P Wendling F Epileptogenicity of brain structures in human temporal lobe epilepsy: a quantified study from intracerebral EEG. Brain (2008) 131(Pt 7):1818–30 10.1093/brain/awn111 18556663

[45] Schindler K Leung H Elger CE Lehnertz K Assessing seizure dynamics by analysing the correlation structure of multichannel intracranial EEG. Brain (2007) 130(Pt 1):65–77 10.1093/brain/awl304 17082199

[46] Ortega GJ Peco IH Sola RG Pastor J Impaired mesial synchronization in temporal lobe epilepsy. Neurophysiol Clin (2011) 122(6):1106–16 10.1016/j.clinph.2010.11.001 21185775

[47] Mankinen K Long XY Paakki JJ Harila M Rytky S Tervonen O Alterations in regional homogeneity of baseline brain activity in pediatric temporal lobe epilepsy. Brain Res (2011) 1373:221–9 10.1016/j.brainres.2010.12.004 21146507

[48] Stufflebeam SM Liu H Sepulcre J Tanaka N Buckner RL Madsen JR Localization of focal epileptic discharges using functional connectivity magnetic resonance imaging. J Neurosurg (2011) 114(6):1693–7 10.3171/2011.1.JNS10482 21351832 PMC3248962

[49] Luo C Li Q Xia Y Lei X Xue K Yao Z Resting state basal ganglia network in idiopathic generalized epilepsy. Hum Brain Mapp (2012) 33(6):1279–94 10.1002/hbm.21286 21520351 PMC6869872

[50] Masterton RA Carney PW Jackson GD Cortical and thalamic resting-state functional connectivity is altered in childhood absence epilepsy. Epilepsy Res (2012) 99(3):327–34 10.1016/j.eplepsyres.2011.12.014 22281064

[51] Wang Z Zhang Z Jiao Q Liao W Chen G Sun K Impairments of thalamic nuclei in idiopathic generalized epilepsy revealed by a study combining morphological and functional connectivity MRI. PLoS One (2012) 7(7):e39701 10.1371/journal.pone.0039701 22808050 PMC3394762

[52] Bai X Guo J Killory B Vestal M Berman R Negishi M Resting functional connectivity between the hemispheres in childhood absence epilepsy. Neurology (2011) 76:1960–7 10.1212/WNL.0b013e31821e54de 21646622 PMC3109878

[53] Moeller F LeVan P Gotman J Independent component analysis (ICA) of generalized spike wave discharges in fMRI: comparison with general linear model-based EEG-fMRI. Hum Brain Mapp (2011) 32(2):209–17 10.1002/hbm.21010 20336659 PMC3753294

[54] Zhong Y Lu G Zhang Z Jiao Q Li K Liu Y Altered regional synchronization in epileptic patients with generalized tonic-clonic seizures. Epilepsy Res (2011) 97(1–2):83–91 10.1016/j.eplepsyres.2011.07.007 21856123

[55] Holmes M Folley BS Sonmezturk HH Gore JC Kang H Abou-Khalil B Resting state functional connectivity of the hippocampus associated with neurocognitive function in left temporal lobe epilepsy. Hum Brain Mapp (2014) 35(3):735–44 10.1002/hbm.22210 23124719 PMC3915042

[56] Kobayashi E Bagshaw AP Benar CG Aghakhani Y Andermann F Dubeau F Temporal and extratemporal BOLD responses to temporal lobe interictal spikes. Epilepsia (2006) 47(2):343–54 10.1111/j.1528-1167.2006.00427.x 16499759

[57] Krakow K Woermann FG Symms MR Allen PJ Lemieux L Barker GJ EEG-triggered functional MRI of interictal epileptiform activity in patients with partial seizures. Brain (1999) 122(Pt 9):1679–88 10.1093/brain/122.9.1679 10468507

[58] Salek-Haddadi A Diehl B Hamandi K Merschhemke M Liston A Friston K Hemodynamic correlates of epileptiform discharges: an EEG-fMRI study of 63 patients with focal epilepsy. Brain Res (2006) 1088(1):148–66 10.1016/j.brainres.2006.02.098 16678803

[59] Thornton R Laufs H Rodionov R Cannadathu S Carmichael DW Vulliemoz S EEG correlated functional MRI and postoperative outcome in focal epilepsy. J Neurol Neurosurg Psychiatry (2010) 81(8):922–7 10.1136/jnnp.2009.196253 20547617

[60] Pittau F Dubeau F Gotman J Contribution of EEG/fMRI to the definition of the epileptic focus. Neurology (2012) 78(19):1479–87 10.1212/WNL.0b013e3182553bf7 22539574 PMC3345614

[61] De Tiege X Laufs H Boyd SG Harkness W Allen PJ Clark CA EEG-fMRI in children with pharmacoresistant focal epilepsy. Epilepsia (2007) 48(2):385–9 10.1111/j.1528-1167.2006.00951.x 17295635

[62] van Houdt PJ de Munck JC Leijten FS Huiskamp GJ Colon AJ Boon PA EEG-fMRI correlation patterns in the presurgical evaluation of focal epilepsy: a comparison with electrocorticographic data and surgical outcome measures. Neuroimage (2013) 75:238–48 10.1016/j.neuroimage.2013.02.033 23454472

[63] Elshoff L Groening K Grouiller F Wiegand G Wolff S Michel C The value of EEG-fMRI and EEG source analysis in the presurgical setup of children with refractory focal epilepsy. Epilepsia (2012) 53(9):1597–606 10.1111/j.1528-1167.2012.03587.x 22779700

[64] Gotman J Grova C Bagshaw A Kobayashi E Aghakhani Y Dubeau F Generalized epileptic discharges show thalamocortical activation and suspension of the default state of the brain. Proc Natl Acad Sci U S A (2005) 102(42):15236–40 10.1073/pnas.0504935102 16217042 PMC1257704

[65] Hamandi K Laufs H Noth U Carmichael DW Duncan JS Lemieux L BOLD and perfusion changes during epileptic generalised spike wave activity. Neuroimage (2008) 39(2):608–18 10.1016/j.neuroimage.2007.07.009 17920297

[66] Hamandi K Salek-Haddadi A Laufs H Liston A Friston K Fish DR EEG-fMRI of idiopathic and secondarily generalized epilepsies. Neuroimage (2006) 31(4):1700–10 10.1016/j.neuroimage.2006.02.016 16624589

[67] Salek-Haddadi A Lemieux L Merschhemke M Friston KJ Duncan JS Fish DR Functional magnetic resonance imaging of human absence seizures. Ann Neurol (2003) 53(5):663–7 10.1002/ana.10586 12731002

[68] Aghakhani Y Bagshaw AP Benar CG Hawco C Andermann F Dubeau F fMRI activation during spike and wave discharges in idiopathic generalized epilepsy. Brain (2004) 127(Pt 5):1127–44 10.1093/brain/awh136 15033899

[69] van Houdt PJ de Munck JC Zijlmans M Huiskamp G Leijten FS Boon PA Comparison of analytical strategies for EEG-correlated fMRI data in patients with epilepsy. Magn Reson Imaging (2010) 28(8):1078–86 10.1016/j.mri.2010.03.022 20471191

[70] Hauf M Jann K Schindler K Scheidegger O Meyer K Rummel C Localizing seizure-onset zones in presurgical evaluation of drug-resistant epilepsy by electroencephalography/fMRI: effectiveness of alternative thresholding strategies. AJNR Am J Neuroradiol (2012) 33(9):1818–24 10.3174/ajnr.A3052 22538072 PMC7964757

[71] Grova C Daunizeau J Lina JM Benar CG Benali H Gotman J Evaluation of EEG localization methods using realistic simulations of interictal spikes. Neuroimage (2006) 29(3):734–53 10.1016/j.neuroimage.2005.08.053 16271483

[72] Vulliemoz S Lemieux L Daunizeau J Michel CM Duncan JS The combination of EEG source imaging and EEG-correlated functional MRI to map epileptic networks. Epilepsia (2010) 51(4):491–505 10.1111/j.1528-1167.2009.02342.x 19817805

[73] Thornton R Vulliemoz S Rodionov R Carmichael DW Chaudhary UJ Diehl B Epileptic networks in focal cortical dysplasia revealed using electroencephalography-functional magnetic resonance imaging. Ann Neurol (2011) 70(5):822–37 10.1002/ana.22535 22162063 PMC3500670

[74] Aubert S Wendling F Regis J McGonigal A Figarella-Branger D Peragut JC Local and remote epileptogenicity in focal cortical dysplasias and neurodevelopmental tumours. Brain (2009) 132(Pt 11):3072–86 10.1093/brain/awp242 19770216

[75] Fauser S Sisodiya SM Martinian L Thom M Gumbinger C Huppertz HJ Multi-focal occurrence of cortical dysplasia in epilepsy patients. Brain (2009) 132(Pt 8):2079–90 10.1093/brain/awp145 19506069

[76] Vaudano AE Carmichael DW Salek-Haddadi A Rampp S Stefan H Lemieux L Networks involved in seizure initiation. A reading epilepsy case studied with EEG-fMRI and MEG. Neurology (2012) 79(3):249–53 10.1212/WNL.0b013e31825fdf3a 22764255 PMC3398433

[77] Murta T Leal A Garrido MI Figueiredo P Dynamic causal modelling of epileptic seizure propagation pathways: a combined EEG-fMRI study. Neuroimage (2012) 62(3):1634–42 10.1016/j.neuroimage.2012.05.053 22634857 PMC3778869

[78] Moeller F LeVan P Muhle H Stephani U Dubeau F Siniatchkin M Absence seizures: individual patterns revealed by EEG-fMRI. Epilepsia (2010) 51(10):2000–10 10.1111/j.1528-1167.2010.02698.x 20726875 PMC3769289

[79] Tyvaert L Chassagnon S Sadikot A LeVan P Dubeau F Gotman J Thalamic nuclei activity in idiopathic generalized epilepsy: an EEG-fMRI study. Neurology (2009) 73(23):2018–22 10.1212/WNL.0b013e3181c55d02 19996076

[80] Daunizeau J Lemieux L Vaudano AE Friston KJ Stephan KE An electrophysiological validation of stochastic DCM for fMRI. Front Comput Neurosci (2012) 6:103 10.3389/fncom.2012.00103 23346055 PMC3548242

[81] Waites AB Briellmann RS Saling MM Abbott DF Jackson GD Functional connectivity networks are disrupted in left temporal lobe epilepsy. Ann Neurol (2006) 59(2):335–43 10.1002/ana.20733 16404743

[82] Zhang Z Lu G Zhong Y Tan Q Liao W Chen Z Impaired perceptual networks in temporal lobe epilepsy revealed by resting fMRI. J Neurol (2009) 256(10):1705–13 10.1007/s00415-009-5187-2 19488674

[83] Zhang Z Lu G Zhong Y Tan Q Yang Z Liao W Impaired attention network in temporal lobe epilepsy: a resting FMRI study. Neurosci Lett (2009) 458(3):97–101 10.1016/j.neulet.2009.04.040 19393717

[84] Maneshi M Moeller F Fahoum F Gotman J Grova C Resting-state connectivity of the sustained attention network correlates with disease duration in idiopathic generalized epilepsy. PLoS One (2012) 7:e50359 10.1371/journal.pone.0050359 23227168 PMC3515589

[85] Wang Z Lu G Zhang Z Zhong Y Jiao Q Tan Q Altered resting state networks in epileptic patients with generalized tonic-clonic seizures. Brain Res (2011) 1374:134–41 10.1016/j.brainres.2010.12.034 21167825

[86] Vollmar C O’Muircheartaigh J Barker GJ Symms MR Thompson P Kumari V Motor system hyperconnectivity in juvenile myoclonic epilepsy: a cognitive functional magnetic resonance imaging study. Brain (2011) 134(Pt 6):1710–9 10.1093/brain/awr098 21616969 PMC3102244

[87] Stretton J Winston GP Sidhu M Bonelli S Centeno M Vollmar C Disrupted segregation of working memory networks in temporal lobe epilepsy. Neuroimage Clin (2013) 2:273–81 10.1016/j.nicl.2013.01.009 24179782 PMC3777779

[88] Chaudhary UJ Centeno M Carmichael DW Vollmar C Rodionov R Bonelli S Imaging the interaction: epileptic discharges, working memory, and behavior. Hum Brain Mapp (2013) 34(11):2910–7 10.1002/hbm.22115 22711681 PMC6870208

[89] Fahoum F Lopes R Pittau F Dubeau F Gotman J Widespread epileptic networks in focal epilepsies: EEG-fMRI study. Epilepsia (2012) 53(9):1618–27 10.1111/j.1528-1167.2012.03533.x 22691174 PMC4492710

[90] Fahoum F Zelmann R Tyvaert L Dubeau F Gotman J Epileptic discharges affect the default mode network – FMRI and intracerebral EEG evidence. PLoS One (2013) 8(6):e68038 10.1371/journal.pone.0068038 23840805 PMC3695970

[91] Carmichael DW Hamandi K Laufs H Duncan JS Thomas DL Lemieux L An investigation of the relationship between BOLD and perfusion signal changes during epileptic generalised spike wave activity. Magn Reson Imaging (2008) 26(7):870–3 10.1016/j.mri.2008.01.041 18501544 PMC2702128

[92] Lui S Ouyang L Chen Q Huang X Tang H Chen H Differential interictal activity of the precuneus/posterior cingulate cortex revealed by resting state functional MRI at 3T in generalized vs. partial seizure. J Magn Reson Imaging (2008) 27(6):1214–20 10.1002/jmri.21370 18504738

[93] Zhang Z Lu G Zhong Y Tan Q Chen H Liao W fMRI study of mesial temporal lobe epilepsy using amplitude of low-frequency fluctuation analysis. Hum Brain Mapp (2010) 31:1851–61 10.1002/hbm.20982 20225278 PMC6870704

[94] Zhang Z Lu G Zhong Y Tan Q Liao W Wang Z Altered spontaneous neuronal activity of the default-mode network in mesial temporal lobe epilepsy. Brain Res (2010) 1323:152–60 10.1016/j.brainres.2010.01.042 20132802

[95] Liao W Zhang Z Pan Z Mantini D Ding J Duan X Altered functional connectivity and small-world in mesial temporal lobe epilepsy. PLoS One (2010) 5(1):e8525 10.1371/journal.pone.0008525 20072616 PMC2799523

[96] Haneef Z Lenartowicz A Yeh HJ Engel J Stern JM Effect of lateralized temporal lobe epilepsy on the default mode network. Epilepsy Behav (2012) 25:350–7 10.1016/j.yebeh.2012.07.019 23103309 PMC4209897

[97] Mankinen K Jalovaara P Paakki JJ Harila M Rytky S Tervonen O Connectivity disruptions in resting-state functional brain networks in children with temporal lobe epilepsy. Epilepsy Res (2012) 100(1–2):168–78 10.1016/j.eplepsyres.2012.02.010 22418271

[98] Widjaja E Zamyadi M Raybaud C Snead OC Smith ML Impaired default mode network on resting-state fMRI in children with medically refractory epilepsy. AJNR Am J Neuroradiol (2013) 34(3):552–7 10.3174/ajnr.A3265 22954741 PMC7964920

[99] McGill ML Devinsky O Kelly C Milham M Castellanos FX Quinn BT Default mode network abnormalities in idiopathic generalized epilepsy. Epilepsy Behav (2012) 23(3):353–9 10.1016/j.yebeh.2012.01.013 22381387 PMC4407647

[100] Yang T Luo C Li Q Guo Z Liu L Gong Q Altered resting-state connectivity during interictal generalized spike-wave discharges in drug-naïve childhood absence epilepsy. Hum Brain Mapp (2012) 34(8):1761–7 10.1002/hbm.22025 22431250 PMC6870260

[101] Luo C Li Q Lai Y Xia Y Qin Y Liao W Altered functional connectivity in default mode network in absence epilepsy: a resting-state fMRI study. Hum Brain Mapp (2011) 32(3):438–49 10.1002/hbm.21034 21319269 PMC6870112

[102] Song M Du H Wu N Hou B Wu G Wang J Impaired resting-state functional integrations within default mode network of generalized tonic-clonic seizures epilepsy. PLoS One (2011) 6:e17294 10.1371/journal.pone.0017294 21364890 PMC3045438

[103] Raichle ME Snyder AZ A default mode of brain function: a brief history of an evolving idea. Neuroimage (2007) 37(4):1083–90 discussion 97-9, 10.1016/j.neuroimage.2007.02.041 17719799

[104] Vaessen MJ Braakman HM Heerink JS Jansen JF Debeij-van Hall MH Hofman PA Abnormal modular organization of functional networks in cognitively impaired children with frontal lobe epilepsy. Cereb Cortex (2012) 23(8):1997–2006 10.1093/cercor/bhs186 22772649

[105] Zhang X Tokoglu F Negishi M Arora J Winstanley S Spencer DD Social network theory applied to resting-state fMRI connectivity data in the identification of epilepsy networks with iterative feature selection. J Neurosci Methods (2011) 199(1):129–39 10.1016/j.jneumeth.2011.04.020 21570425 PMC3129815

[106] Zhang Z Liao W Chen H Mantini D Ding JR Xu Q Altered functional-structural coupling of large-scale brain networks in idiopathic generalized epilepsy. Brain (2011) 134(Pt 10):2912–28 10.1093/brain/awr223 21975588

[107] Yao Z Zhang Y Lin L Zhou Y Xu C Jiang T Abnormal cortical networks in mild cognitive impairment and Alzheimer’s disease. PLoS Comput Biol (2010) 6(11):e1001006 10.1371/journal.pcbi.1001006 21124954 PMC2987916

[108] Rodionov R De Martino F Laufs H Carmichael DW Formisano E Walker M Independent component analysis of interictal fMRI in focal epilepsy: comparison with general linear model-based EEG-correlated fMRI. Neuroimage (2007) 38(3):488–500 10.1016/j.neuroimage.2007.08.003 17889566

[109] Carmichael DW Vulliemoz S Rodionov R Thornton JS McEvoy AW Lemieux L Simultaneous intracranial EEG-fMRI in humans: protocol considerations and data quality. Neuroimage (2012) 63(1):301–9 10.1016/j.neuroimage.2012.05.056 22652020

[110] Vulliemoz S Carmichael DW Rosenkranz K Diehl B Rodionov R Walker MC Simultaneous intracranial EEG and fMRI of interictal epileptic discharges in humans. Neuroimage (2011) 54(1):182–90 10.1016/j.neuroimage.2010.08.004 20708083

[111] Grouiller F Thornton RC Groening K Spinelli L Duncan JS Schaller K With or without spikes: localization of focal epileptic activity by simultaneous electroencephalography and functional magnetic resonance imaging. Brain (2011) 134(Pt 10):2867–86 10.1093/brain/awr156 21752790 PMC3656675

[112] Britz J Van De Ville D Michel CM BOLD correlates of EEG topography reveal rapid resting-state network dynamics. Neuroimage (2010) 52(4):1162–70 10.1016/j.neuroimage.2010.02.052 20188188

[113] Yuan H Zotev V Phillips R Drevets WC Bodurka J Spatiotemporal dynamics of the brain at rest – exploring EEG microstates as electrophysiological signatures of BOLD resting state networks. Neuroimage (2012) 60(4):2062–72 10.1016/j.neuroimage.2012.02.031 22381593

[114] Kay BP DiFrancesco MW Privitera MD Gotman J Holland SK Szaflarski JP Reduced default mode network connectivity in treatment-resistant idiopathic generalized epilepsy. Epilepsia (2013) 54(3):461–70 10.1111/epi.12057 23293853 PMC3593969

[115] Holmes MJ Yang X Landman Ba Ding Z Abou-Khalil B Sonmezturk HH Functional networks in temporal lobe epilepsy: a voxel-wise study of resting state functional connectivity and gray matter concentration. Brain Connect (2013) 3(1):22–30 10.1089/brain.2012.0103 23150897 PMC3621340

[116] Doucet G Osipowicz K Sharan A Sperling MR Tracy JI Extratemporal functional connectivity impairments at rest are related to memory performance in mesial temporal epilepsy. Hum Brain Mapp (2013) 34(9):2202–16 10.1002/hbm.22059 22505284 PMC3864618

[117] Negishi M Martuzzi R Novotny EJ Spencer DD Constable RT Functional MRI connectivity as a predictor of the surgical outcome of epilepsy. Epilepsia (2011) 52:1733–40 10.1111/j.1528-1167.2011.03191.x 21801165 PMC3169719

[118] Christodoulou JA Walker LM Del Tufo SN Katzir T Gabrieli JD Whitfield-Gabrieli S Abnormal structural and functional brain connectivity in gray matter heterotopia. Epilepsia (2012) 53(6):1024–32 10.1111/j.1528-1167.2012.03466.x 22524972 PMC3370071

[119] Liao W Zhang Z Pan Z Mantini D Ding J Duan X Default mode network abnormalities in mesial temporal lobe epilepsy: a study combining fMRI and DTI. Hum Brain Mapp (2011) 32(6):883–95 10.1002/hbm.21076 20533558 PMC6870458

